# Application of the Movable Type Free Energy Method to the Caspase-Inhibitor Binding Affinity Study

**DOI:** 10.3390/ijms20194850

**Published:** 2019-09-29

**Authors:** Song Xue, Hao Liu, Zheng Zheng

**Affiliations:** 1 School of Statistics and Mathematics, Zhongnan University of Law and Economics, Wuhan 430073, China; songxue@outlook.com; 2 School of Mechanical and Electronic Engineering, Wuhan University of Technology, Wuhan 430070, China; haoliulh@gmail.com; 3 School of Chemistry, Chemical Engineering and Life Science, Wuhan University of Technology, Wuhan 430070, China

**Keywords:** caspase inhibition, protein-ligand binding free energy, Monte Carlo sampling, docking and scoring, molecular conformational sampling

## Abstract

Many studies have provided evidence suggesting that caspases not only contribute to the neurodegeneration associated with Alzheimer’s disease (AD) but also play essential roles in promoting the underlying pathology of this disease. Studies regarding the caspase inhibition draw researchers’ attention through time due to its therapeutic value in the treatment of AD. In this work, we apply the “Movable Type” (MT) free energy method, a Monte Carlo sampling method extrapolating the binding free energy by simulating the partition functions for both free-state and bound-state protein and ligand configurations, to the caspase-inhibitor binding affinity study. Two test benchmarks are introduced to examine the robustness and sensitivity of the MT method concerning the caspase inhibition complexing. The first benchmark employs a large-scale test set including more than a hundred active inhibitors binding to caspase-3. The second benchmark includes several smaller test sets studying the relative binding free energy differences for minor structural changes at the caspase-inhibitor interaction interfaces. Calculation results show that the RMS errors for all test sets are below 1.5 kcal/mol compared to the experimental binding affinity values, demonstrating good performance in simulating the caspase-inhibitor complexing. For better understanding the protein-ligand interaction mechanism, we then take a closer look at the global minimum binding modes and free-state ligand conformations to study two pairs of caspase-inhibitor complexes with (1) different caspase targets binding to the same inhibitor, and (2) different polypeptide inhibitors targeting the same caspase target. By comparing the contact maps at the binding site of different complexes, we revealed how small structural changes affect the caspase-inhibitor interaction energies. Overall, this work provides a new free energy approach for studying the caspase inhibition, with structural insight revealed for both free-state and bound-state molecular configurations.

## 1. Introduction

Alzheimer disease (AD) is a neurodegenerative disorder characterized by the neuronal and synaptic loss as well as the accumulation of β-amyloid plaques and neurofibrillary tangles (NFTs) within selective brain regions. Yet its cause, time course or mechanisms are still not well understood [1,2,3,4]. Scientists have proven that the programmed cell death pathway, also known as apoptosis, plays a significant role in the pathogenesis of age-related neurodegenerative diseases, particularly in AD [2,5,6,7]. Caspases, a family of serine-aspartyl proteases, are involved in the initiation and execution of apoptosis. They are known to exist in our cells as inactive precursors which kill the cell once activated and lead to the proteolytic cleavage of several neuronal proteins including tau, APP, presenilin (PS1, PS2), actin, fodrin, etc. Therefore, caspases are believed to be critically related to the pathogenesis of AD [4,8,9,10,11]. Many research results have been published to elucidate the correlation between AD pathogenesis and caspases family members, mostly caspases-2, 3, 6, 7, 8 and 9 [2,12,13,14,15,16,17,18,19]. These studies suggest that preventing caspase activation may be a promising therapeutic for the treatment of AD. The activation or the activity of the caspases can be regulated in two ways: (1) specific molecules such as Bcl-2, FLIP or IAPs can be used to control the processing and activation of a caspase; (2) a number of molecules that directly interact with a caspase can be used to inhibit the proteases that have already been activated. These molecules are called caspase inhibitors [20,21,22,23,24]. Various caspase inhibitors, including small molecules, peptidomimetic and peptide compounds, have been designed to study the relationship between caspases and other factors involved in apoptosis.

Structure-based drug design using high-performance computers have long played important roles in the de novo drug/biomolecule discovery studies. The long-pursued essential of structure-based drug design is the estimation of the free energy change associated with the binding process of a ligand to a biochemical system, for which the calculation speed and accuracy are both crucial [25,26,27]. A number of free energy estimation methods have been developed, including end-point methods, pathway-based free energy calculations, and pathway-independent free energy methods. The end-point methods for free energy estimation (e.g., docking, molecular mechanics combined with the Poisson−Boltzmann or generalized Born and surface area continuum solvation (MMPBSA or MMGBSA)) are relatively fast, but the single static structure which they usually rely on often leads to the neglect of the receptor flexibility and thus compromise the calculation accuracy [28,29,30,31]. The pathway-based free energy methods, which can be broadly categorized into alchemical and potential of mean force approaches, are usually computationally expensive due to the extensive sampling required to estimate the binding free energies [32,33,34]. The alchemical approaches use the thermodynamic cycle built with nonphysical intermediate states to compute the free energy differences between the end states. The two most commonly used alchemical free energy methods are Free energy perturbation (FEP) [35] and thermodynamic integration (TI) [36]. The potential of mean force (PMF) approach [37,38,39], with umbrella sampling coupled with the WHAM (weighted histogram analysis method) analysis, is one of the most widely adopted PMF approaches [40]. Other than the high computational cost caused by the intensive sampling, the pathway free energy methods are also limited by simulation time scales. Constitutionally, the underlying force field has a powerful hold on the accuracy of all free energy estimation methods, leaving improvements in all these methods an active area of research [41,42,43]. On the other hand, the pathway-independent free energy methods, e.g., Monte Carlo free energy sampling methods, use Markov model for the molecular configurational-state sampling. Such methods could potentially gain significant speed benefits from parallel computing according to their stochastic sampling protocols, which also avoids the difficulty of crossing the energy barriers during simulations in the pathway-based methods. However, to generate a converged energy ensemble takes no less computational effort compared to the pathway-based methods. Capturing the significant configurational states are crucial for the pathway-independent free energy methods, which require thorough and careful sampling against the energy landscape.

In this research, we used the Movable Type (MT) free energy method, a novel Monte Carlo free energy algorithm developed by our group to evaluate the binding affinity between a variety of caspase inhibitors and their caspase targets [44]. By comparing the binding free energies and the predicted significant binding modes calculated by our simulation model to those obtained from experiments, we could validate the accuracy of our model against this particular protein target family, and provide potential theoretical support for the future development of the therapeutic intervention for AD.

## 2. Results and Discussion

The goals of this research are (1) to examine the accuracy of Movable Type free energy method in calculating the binding free energy between different caspase targets and various inhibitors, and (2) to apply the MT method to the structural analysis of the caspase-inhibitor binding mechanism. The results could provide theoretical support to proceed further study the feasibility of applying the Movable Type Free Energy Method to design caspase ligand inhibitors, which are closely related to Alzheimer’s disease.

Two different test benchmarks were introduced in this work. First, a relatively large test set was studied to obtain a general picture of the MT method’s performance to differentiate the binding affinities of a large variety of ligand structures binding to the caspase-3 protein target (the caspase target having the most significant number of ligands with known binding affinities). Then we performed a series of relative binding free energy reproduction studies to carefully examine the MT binding affinity prediction regarding (1) ligands of different structural categories bound to specific targets, and (2) ligands from the same structural category bound to different caspase targets, for more detailed computational study of the caspase-ligand bindings.

### 2.1. Large-Scale Validation Benchmark

The first benchmark includes structures and IC50 data (which can be converted to binding free energy via approximation) of 113 small molecular ligands bound to the caspase-3 target that are proven to have binding affinities, published on DUD-E data website (http://dude.docking.org/). The DUD-E website provides several hundred structures of small molecules that actively bind to caspase-3. After screening, redundancy structures, as well as structures with high molecular weights (MW) (>1000 Da) or high degrees of freedom (>1000 rotatable bonds) were abandoned, with the rest 113 active ligands forming the test set used in our validation. Ligand structures were prepared by adding the missing hydrogen atoms, Missing residues at the caspase-3 target protein were added and locally optimized before the calculation. The active compounds’ IC50 data collected from the DUD-E website were transferred to pIC50 values and further approximated to the binding free energies by assigning the unit of energy:

(1) $$\Delta G_{binding}=RT\ln K_{d}\approx RT\ln IC50=-RT\times \frac{e}{10}\times pIC50$$

where *R* is the gas constant and *T* is temperature in Kelvin, which is set to 298.15 K in this work; *e* is the base of the natural logarithm.

IC50 is strongly related to the inhibitor’s binding affinity, and also affected by other factors as the substrate’s and receptor’s concentrations. The inhibitor’s binding affinities can be approximated as pIC50 values when the substrate’s concentration is very small. On one hand, IC50 data are more easily accessible compared to *K*_i_ or *K*_d_ data from the public databases [45], being popular for the large-scaling binding affinity prediction evaluations provided by many widely used databases like BindingDB [45,46], DUD-E [47] and ChEMBL [48], etc. On the other hand, not all experimental IC50 values are comparable to the binding affinity data if without small enough substrate concentrations [49], plus that different experimental IC50 values have been found regarding the same protein-ligand complex system [50], indicating reliability issues for using the public databases in the calculation evaluations. Despite the aforementioned issues, IC50 data are still broadly used in the virtual screening and binding affinity simulation studies [51,52,53], partly because of the limited accessible *K*_i_ or *K*_d_ data, and also because the substrate’s or receptor’s concentration-related terms can be cancelled out (Equation (2)) when comparing the relative binding affinities of those protein-ligand complex systems with the same mechanism of inhibition e.g., virtual screening study targeting the same receptor’s binding site (Equation (3)).

(2) $$\frac{K_{i,1}}{K_{i,2}}=\frac{IC_{50,}{}_{1}}{IC_{50,}{}_{2}}$$

(3) $$\Delta \Delta G_{1,2}=\Delta G_{1}-\Delta G_{2}=\left(-RT\ln K_{i,1}\right)-\left(-RT\ln K_{i,2}\right)=\left(-RT\ln IC_{50,}{}_{1}\right)-\left(-RT\ln IC_{50,}{}_{2}\right)$$

**Figure 1:**
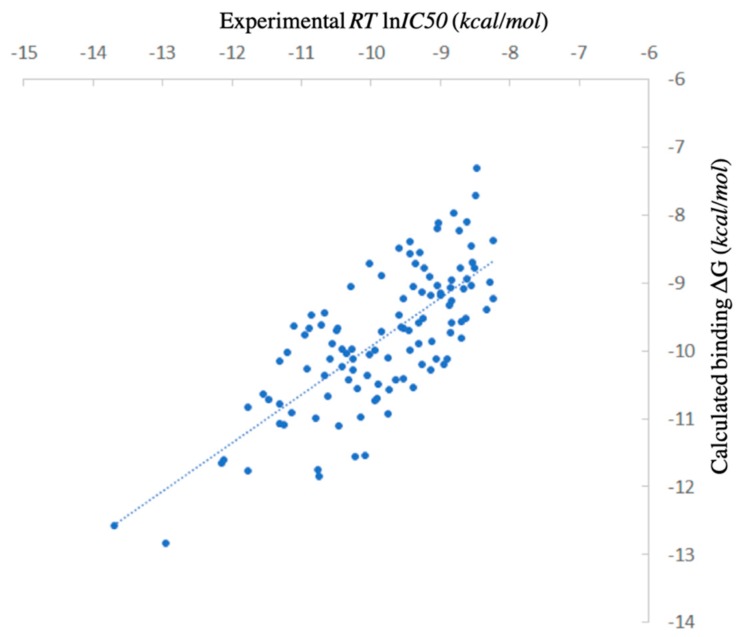
Scattered plot comparing binding free energy calculated by Movable Type Method to experimental data for the DUD-E CASP3 test set (Table S1).

The MT protocol was utilized to perform the virtual screening. The calculation results were shown in Figure 1 together with the experimental *R*Tln*IC50* data generated using Equation (1). As active compounds, all the ligands in this test set are relatively tight binders, with the binding affinity distributed between −8 to −14 kcal/mol and mostly ranged between −8 to −12 kcal/mol. Statistics of this calculation approach showed an RMSE as 0.746 kcal/mol, the *r*^2^ coefficient as 0.552 and Kendall’s tau correlation as 0.506, revealing a good prediction accuracy and ranking capability of the MT method against the large-scaling caspase-3 target-ligand virtual screening test set (Figure 1). Introducing the first test benchmark revealed a general picture of the binding affinity prediction using the MT method against a large number of active small molecules, with diverse structural features, bound to the caspase-3 target. Further explorations including relative binding affinity difference study referencing minor structural changes and structural based protein-ligand interaction interface analysis were also carried out to examine the reliability of the MT protocol against the caspase-ligand binding prediction.

### 2.2. Test Benchmark Studying the Binding ∆∆G Regarding the Structural Changes at the Binding Interface

In the second test benchmark, we employed a series of smaller test sets with high quality protein-ligand crystal structures, and carefully categorized ligands according to their structural similarities, so that we can further explore the binding affinity prediction accuracy by using the MT method, its sensitivity against local structural changes at the protein-ligand interaction interfaces, and even more, the potency of applying this method to the inverse docking study related to the caspase inhibitors.

In this test benchmark, protein-ligand complex crystal structures were selected from the Protein Data Bank (PDB) and categorized into three test sets according to the ligand structural features. The first test set aimed to study the relative binding free energy changes of different ligands bound to the same protein target. Given the same target and same binding site residue environment, it was important to explore the capability of the MT method to differentiate the binding affinity against minor to major changes concerning the ligand structures. Caspase-3, as one of the most important AD related target, was selected as the protein target in this test benchmark as well for the relative binding affinity study.

**Figure 2:**
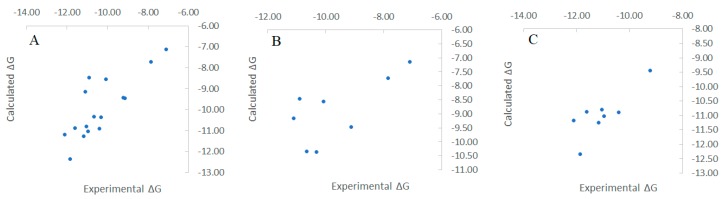
Scattered plots comparing the binding free energy calculated by Moveable Type to experimental data for the caspase-3-Inhibitor test set. (**A**) All the test cases. (**B**) small molecule inhibitors sub-group. (**C**) peptidomimetic inhibitor sub-group.

16 caspase-3 inhibitors were selected from the Protein Data Bank and categorized into two sub-groups based on their structural characteristics: inhibitors with no amino acid structures while having MWs less than 500 Da were selected to the small molecule inhibitors sub-group; inhibitors containing polypeptide backbones with natural or unnatural amino acids were classified to the peptidomimetic inhibitors sub-group. The results of applying the MT method to calculate the binding free energy were listed in Table 1 and Table 2 below:

The small molecule subgroup contains ligands with more spread-out binding affinities while inhibitors in the peptidomimetic inhibitor subgroups are all tight binders. Binding affinity predictions using the MT method were illustrated in Figure 2 to compare with the experimental data. Against the small molecule subgroup, the MT method reproduced an RMSE as 1.242 kcal/mol, *r*^2^ correlation coefficient as 0.501, and Kendall’s tau as 0.357. Regarding the peptidomimetic inhibitor subgroup, the MT calculation results had an RMSE as 0.479 kcal/mol, *r*^2^ coefficient as 0.655, and Kendall’s tau as 0.444 compared to the experimental data. Calculation against the peptidomimetic inhibitor subgroup were generally better than the small molecule subgroup. By merging the two subgroups, we also looked at the MT calculation performance against the total caspase3-ligands test set. For all the 16 different ligands bound to the caspase3 target, we generated an RMSE as 0.920 kcal/mol, *r*^2^ coefficient as 0.647, and Kendall’s tau as 0.559.

In the caspase-3-Inhibitor test set, the ligands’ MWs varied from 301.09 to 838.94 Da, with an *r*^2^ correlation as 0.314 with the binding affinity distribution, compared to the MT calculation results whose *r*^2^ coefficient as 0.647 regarding the experimental data. The MT method is not ligands’ MW dependent, according to this validation. Regarding this test set, the absolute errors of all the MT calculation results were lower than 2.5 kcal/mol for all the 16 complexes, 15 predictions had the absolute errors lower than 2 kcal/mol; 13 predictions had the absolute errors lower than 1 kcal/mol. A generally good binding affinity prediction against the caspase-3-Inhibitor test set were revealed by using the MT free energy protocol.

Hereby we used one example, namely 1gfw, to illustrate the sampled significant ligand’s conformations in the free state and the docked poses in bound state, and the calculated ensemble energies in both free and bound state, to further demonstrate how the MT computational protocol worked.

**Figure 3:**
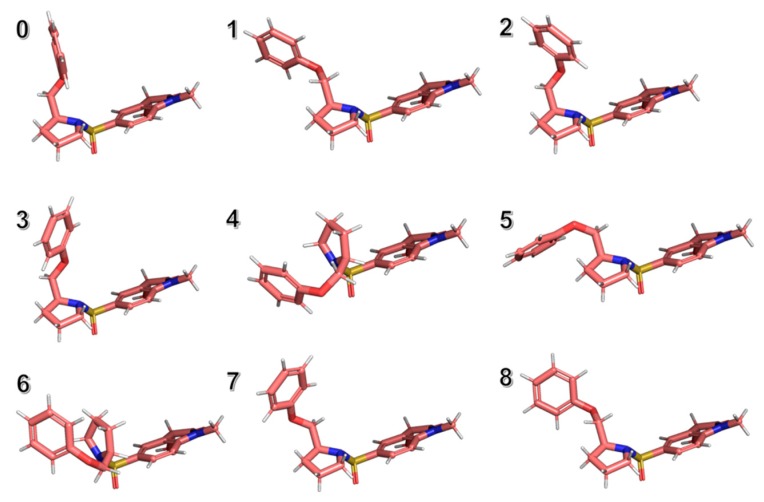
The top 9 significant ligand’s conformations for the ligand in its free solution phase with respect to the 1gfw caspase-3-Inhibitor complex (from conformer 0 to conformer 9), generated by using the MT protocol.

**Figure 4:**
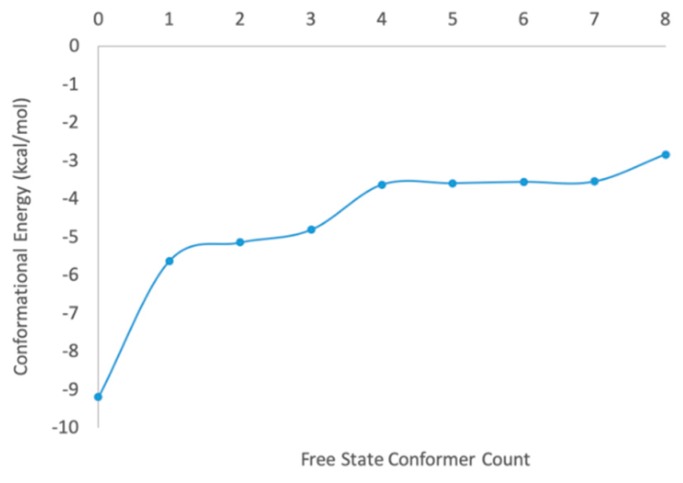
Energy distribution of the top 9 sampled ligand conformations ranked based on the conformational energy in the solution phase.

1gfw contains a relatively small ligand with 5 heavy-heavy atom rotatable bonds. The MT-CS conformational search program generated 134 distinguished conformers and calculated their conformational energies in the solution phase by employing the KMTSIM solvation model. The top 9 ligand conformers according to their energy ranking were shown in Figure 3, with their energy distribution shown in Figure 4. The free-state ligand’s partition function, ZL was in-turn calculated using Equation (6). ZL was a very big number as the sum of all the ligand’s conformational local partition functions, which was shown as −RTlog(ZL) in this work for better revealing its physical meaning. The MT-CS calculation had −RTlog(ZL) = −3.99 kcal/mol, representing the ensemble energy of the free-state ligand’s conformations, an energy barrier that the binding process had to overcome.

**Figure 5:**
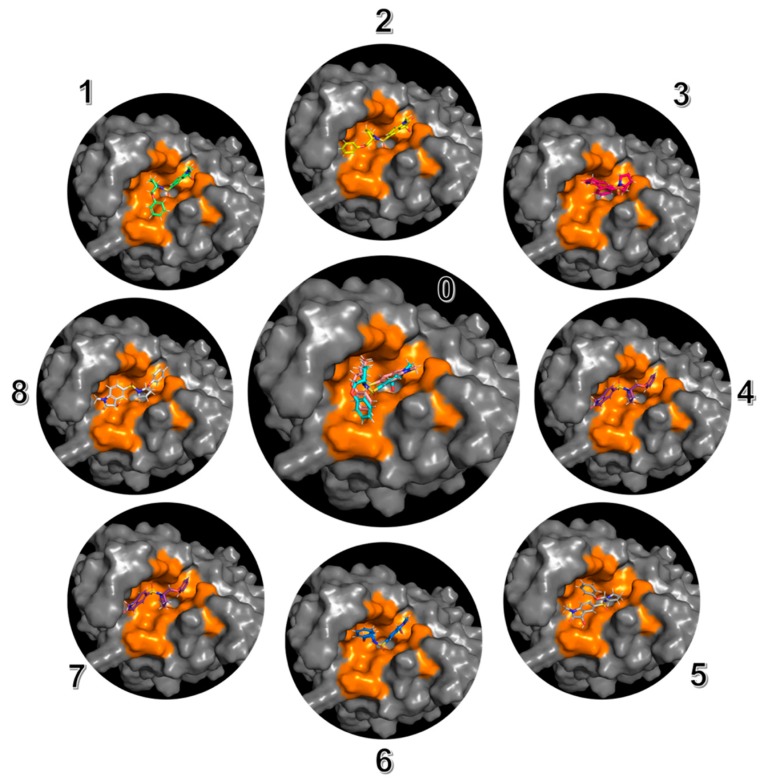
The top 9 significant binding modes for the 1gfw caspase-3-inhibitor complex, indexed from 0 to 9. All the binding modes are generated by using the heatmap docking method. The best docked ligand pose (in cyan) is shown together with the crystal ligand (in pink) in Figure 5-0 in the middle, with a structural RMSD as 2.08 Å.

**Figure 6:**
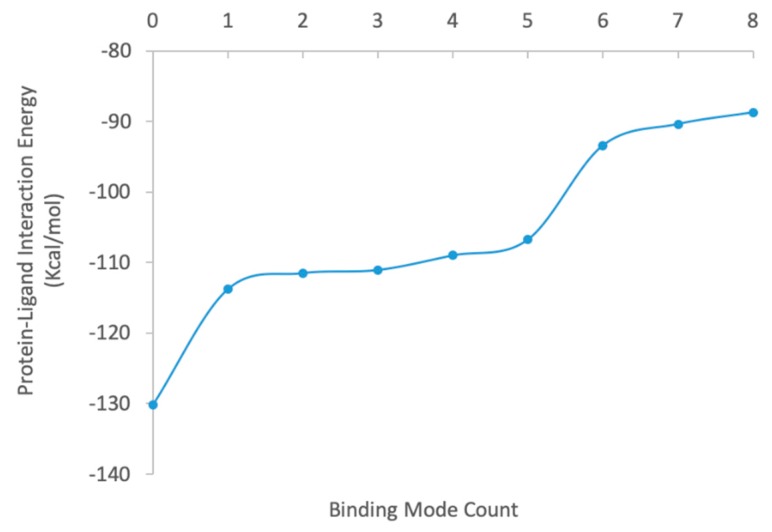
Protein-ligand nonbonding interaction energy distribution of the top 9 sampled binding modes ranked based on the protein-ligand nonbonding interaction energy.

The heatmap docking method generated 115 unique docked poses for this protein-ligand complex. The best docked ligand pose had a structural RMSD as 2.08 Å compared to the ligand’s crystal structure. We showed the top 9 docked complex poses in Figure 5, and the protein-ligand binding interaction energies in Figure 6. ZPL was calculated using Equation (7) summing all the complexes’ configurational local partition functions. −RTlog(ZPL) = −14.33 kcal/mol was generated as the ensemble energy of the complex considering all the 115 binding conformations in the solution phase. So that we derived the final binding free energy using Equation (8). The $\Delta G_{binding}$ was then calculated as −14.33 kcal/mol − (−3.99 kcal/mol) = −10.34 kcal/mol.

Given the success of the first test set, we were encouraged to expand our study on other caspase targets. Polypeptide inhibitors were found with better selectivity and more effective compared to the small molecular inhibitors against the caspase targets, which gradually drew researchers’ attention through time. In this work, we studied the polypeptide inhibitors with similar structures binding to different caspase targets, to explore the performance of the MT method reproducing the small relative binding affinity differences among the test cases.

We collected the crystal structures and binding affinity data of 15 different caspase-polypeptide inhibitor complexes from the Protein Data Bank. The MT protocol was applied to reproduce the binding affinities and significant binding modes reproductions. The calculation results agreed quite well with the experimental data and generated a RMSE as 0.733 kcal/mol, an *r*^2^ coefficient as 0.752, and a Kendall’s tau as 0.651 (Table 3). In the first and second test benchmarks, we focused on different ligands binding to the same caspase target. Within this test set, we particularly examined the cases with the same inhibitor binding to different caspase targets.

Hereby we looked at two pairs of complex structures as representative examples, to examine how the small structural differences at the binding interfaces affecting the binding affinities between the caspase targets and polypeptide inhibitors.

First, we compared the calculation results between 2h5i and 1f1j, two complexes with the same peptide ligand, Ac-DEVD-Cho, targeting different caspase receptors, caspase-3, and caspase-7. The global minimum binding modes for both of the complexes provided us a clear view of their protein-ligand interaction maps. By using the MT protocol, the global minimum binding mode for the caspase-3-Ac-DEVD-Cho complex had a structural RMSD as 1.17 Å, and the global minimum binding mode for the caspase-7-Ac-DEVD-Cho complex had a structural RMSD as 1.44 Å, both compared to their corresponding crystal structures.

**Figure 7:**
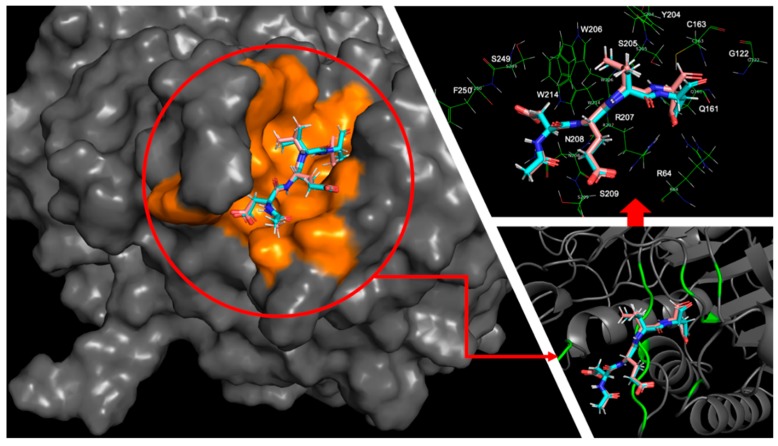
The global minimum docked pose (cyan) together with the crystal ligand conformation (pink) for Ac-DEVD-Cho bound to caspase-3. The orange surface on the left shows the area of interaction interface at the caspase-3 binding site. The green ribbon on the bottom right shows the locations of the residues having close contact (within 3 Å) with the global minimum docked pose. All residues having close contact with the global minimum Ac-DEVD-Cho pose are shown in the picture on the top right.

**Figure 8:**
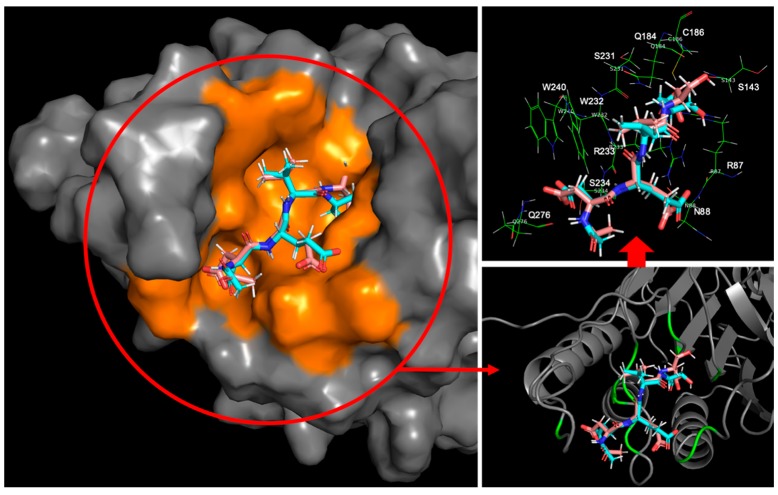
The global minimum docked pose (cyan) together with the crystal ligand conformation (pink) for Ac-DEVD-Cho bound to caspase-7. The orange surface on the left shows the area of interaction interface at the caspase-7 binding site. The green ribbon on the bottom right shows the locations of the residues having close contact (within 3 Å) with the global minimum docked pose. All residues having close contact with the global minimum Ac-DEVD-Cho pose are shown in the picture on the top right.

Both caspase-3 and caspase-7 targets had clip-shaped binding sites with similar volumes occupied by the polypeptide inhibitor, Ac-DEVD-Cho, according to the highlighted area in Figure 7 and Figure 8. Both binding sites used short amino acid chains to form a series of backbone-backbone hydrogen bonds stabilizing the polypeptide inhibitor, i.e., S205, R207 and S209 at the caspase-3 binding site formed 4 hydrogen bonds with the Asp, Val, Glu backbone residues and the acetyl capping group of the polypeptide inhibitor respectively; S231, R233, and Q276 forms 4 hydrogen bonds with the Asp, Val, Glu, and Asp backbone residues as well. W206 and Y204 from caspase-3 applied bulky aromatic side-chain structures to limit the flexibility of the polypeptide inhibitor by holding its Valine side chain in between. Similarly, caspase-7 used the indole side chain of W232 and the phenol side chain of Y230 to drag the ligand’s valine side chain by forming a C-H/π interaction. Several other residues at the protein’s clip-shaped binding site also stabilize the target-inhibitor complex by forming hydrogen bonds with the side chain and capping groups of Ac-DEVD-Cho. At the caspase-3 binding site, W214, S249, and N208 formed hydrogen bonds with the carboxyl side chain of the acetyl capped aspartic acid residue; R207 formed a hydrogen bond with the carboxyl group from the glutamic acid side chain; R64, Q161 and R207 formed hydrogen bonds with the aldehyde capped aspartic acid side chain; and G122 formed a hydrogen bond with the aldehyde capping group on the ligand. On the other hand, at the caspase-7 binding site, S234, W240 and Q276 formed hydrogen bonds with the carboxyl side chain of the inhibitor’s acetyl capped aspartic acid residue; N88 formed a hydrogen bond with the carboxyl group from the glutamic acid side chain; R87, Q184 and R233 formed hydrogen bonds with the aldehyde capped aspartic acid side chain; and R87 also formed a hydrogen bond with the aldehyde capping group on the ligand.

With quite similar interaction maps, the MT protocol generated very close protein-ligand interaction energies of these two global-minimum binding modes. The caspase-3-Ac-DEVD-Cho binding mode had −163.92 kcal/mol for the protein-ligand interface contact energy and the caspase-7-Ac-DEVD-Cho binding mode had −159.73 kcal/mol as its own. It also led to quite similar binding affinity predictions, with −11.18 kcal/mol for 2h5i and −10.74 kcal/mol for 1f1j.

**Figure 9:**
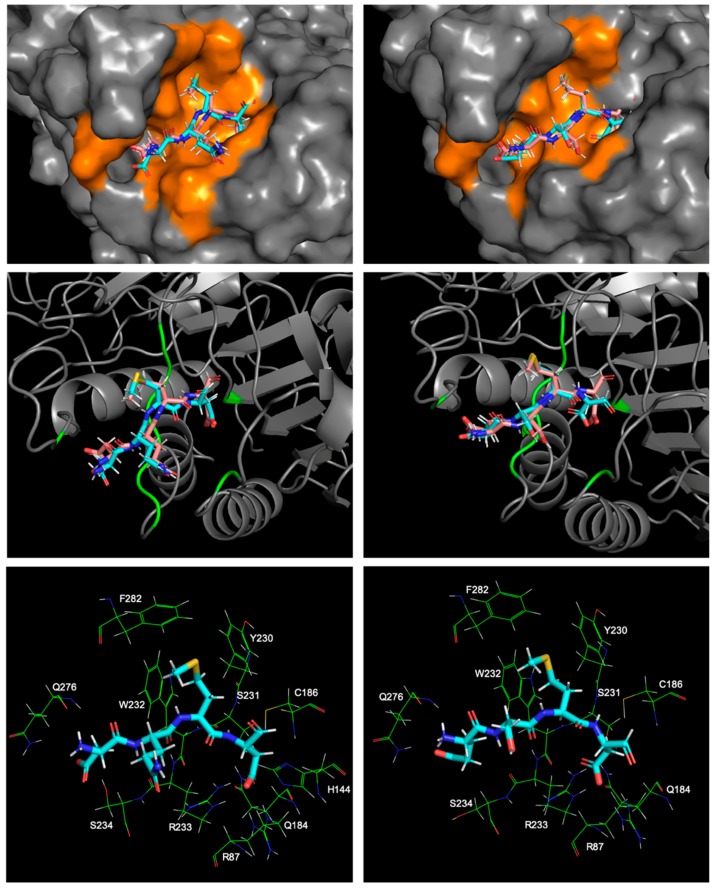
Illustrations of the caspase-7 binding site with Ac-DQMD-Cho (pictures on the left) and Ac-ESMD-Cho (pictures on the right). The global minimum docked pose (cyan) are shown together with the crystal ligand conformation (pink). Orange regions on both binding sites shows the difference of the contacts areas. The green ribbons also indicate more residues from the caspase-7 binding site having significant contact with Ac-DQMD-Cho compared to Ac-ESMD-Cho. Pictures on the bottom show that the glutamine residue from Ac-DQMD-Cho forms extra hydrogen bonds to the R233 residue at the caspase-7 binding site, while no hydrogen bond can be found at the same location for the caspase-7-Ac-ESMD-Cho complex.

Another comparison study focused on the two complexes with the PDBID 2ql9 and 2qlb, using the same target protein: caspase-7, binding to two different polypeptide inhibitors: Ac-DQMD-Cho and Ac-ESMD-Cho. Similarly, in both cases, the caspase target provided a short amino acid chain to seize the peptide inhibitor by a series of hydrogen bonds. S231, W232, R233 and S234 formed four hydrogen bonds with both of the peptide inhibitors’ backbone structures respectively. Also, the caspase-7 receptor prepared Y230, W232 and F282 with their aromatic side chains to stabilize the two inhibitors with the C-H/π interactions. Meanwhile, by introducing the R87, Q184 residues to form hydrogen bonds with the carboxyl groups from the glutamic acid, and the aldehyde capping groups respectively, and by using the Q276 residue to form a hydrogen bond with the acetyl capping groups, the caspase-7 receptor further locked both of the peptide inhibitors at the binding site (Figure 9).

The main reason causing the interaction energy difference for the two inhibitors lay in that the glutamine residue from Ac-DQMD-Cho formed two more hydrogen bonds with the amide group on the R233 residue from the caspase-7 binding site. On the other hand, the side chain of the serine from Ac-ESMD-Cho was too short to stretch out to form such hydrogen bonds. It resulted in the ~10 kcal/mol interaction energy difference between these two global minimum binding modes, with the protein-ligand contact energy as −201.01 kcal/mol for 2ql9 and that as −190.78 kcal/mol for 2qlb. On the other hand, the free-state ligand’s ensemble energy for Ac-DQMD-Cho was −12.217 kcal/mol and that for Ac-ESMD-Cho was −9.18 kcal/mol. It showed that Ac-DQMD-Cho was slightly more favored in the water-solvated free state than Ac-ESMD-Cho, also indicating that the more flexible structure of Ac-DQMD-Cho restored larger configurational entropy compared to Ac-ESMD-Cho. However, the slightly increased protein-ligand complexing barrier for Ac-DQMD-Cho did not stop it from earning ~4 kcal/mol more preferred binding free energy compared to Ac-ESMD-Cho.

## 3. Materials and Methods

The MT method was first developed in our lab in 2013 [44]. Further refinement was later on published in 2018 [54]. Since the detailed illustrations, thorough validations and calculation comparisons with other top-notch methods can be found in our previous publications, and our focus in this work is the MT method validation and application regarding the caspase inhibition instead of a methodology demonstration, only a brief introduction of this method was included in this paper.

The MT method simplifies the molecular energy state simulation and reduces the computational complexity by separating the sampling of the molecular states into samplings of independent atom pairwise contacts during molecular movements. In a molecular system, each atom possesses independent degree of freedom for its movement, hence the free energy change of a molecule can be simulated using the free energy changes of all the atoms in this molecular system. Given that all atoms are allowed a small movement range, the MT method assumes that every pairwise work on atom *A* from another atom *i* is independent from each other. Since every atom, including atom *A* and every atom *i*, possesses its own moving degrees of freedom, all the atom *A*-*i* pairwise energy states can be extrapolated using the EAi vector, where $\tau_{Ai}^{0}$ represents the atom *A*-*i* relative coordinate from the input structure, and Δ*τ* is their geometric deviation step unit with a sampling range (±nΔ*τ*).

(4) EAi=[EAi(τAi0−nΔτ)⋮EAi(τAi0+Δτ)EAi(τAi0)EAi(τAi0−Δτ)⋮EAi(τAi0−nΔτ)]

All energy states for atom *A* from the atom *A*-*i* relative coordinate change can be generated using the reversible work on atom *A* from atom *i* during their movement. The atom pairwise reversible work is calculated as the sum of the work on three orthogonal directions (x, y and z directions) with respect to all the atom *A-i* pairwise energy changes. Equation (5) illustrated the calculation of the pairwise reversible work regarding atom *A* along the *x* axis.

**Figure 10:**
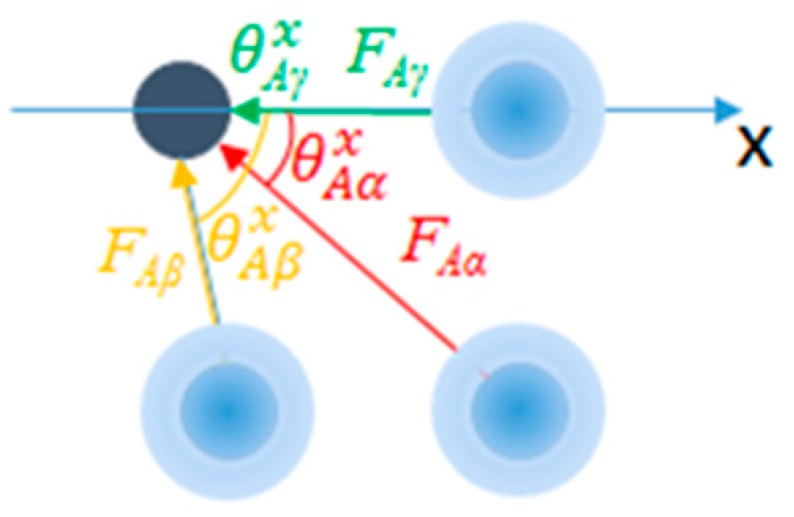
With respect to atom A (dark blue dot on the upper left corner), all atom pairwise contacts on this atom are independent from each other. With atom A moving along the x axis, the ensemble energy.

(5) EAx=∑iN−1FAi×cos(θAi)Δr  =([FAα(rAα1)FAα(rAα2)⋮FAα(rAαn)]×[cos(θAα1)cos(θAα2)⋮cos(θAαn)]+[FAβ(rAβ1)FAβ(rAβ2)⋮FAβ(rAβn)]×[cos(θAβ1)cos(θAβ2)⋮cos(θAβn)]+[FAγ(rAγ1)FAγ(rAγ2)⋮FAγ(rAγn)]×[cos(θAγ1)cos(θAγ2)⋮cos(θAγn)]…)Δr

where FAi represents the vector of forces regarding atom *A-i* with their pairwise distances ranged from $r_{Ai}^{1}$ to $r_{Ai}^{n}$; θAi is the collection of angles of inclination of all the *i*-*A* vector (Figure 10) regarding the x axis. Δr is the sampling step unit. FAi×cos(θAi)Δr generates all the atom *A-i* pairwise energy states. EAx is the ensemble of work on atom *A* from all the surrounding atoms along the x axis.

In this work, we set the distance sampling range ($r_{Ai}^{1}$ to $r_{Ai}^{n}$) as 1 Å, angle sampling range ($\theta_{Ai}^{1}$ to $\theta_{Ai}^{n}$) as 30 degrees, and Δr as 0.005 Å. The reversible work on atom *A* is calculated in all the three orthogonal directions and summed as in Equation (6).

(6) EA=EAx+EAy+EAz

The partition function (*Z*) for atom *A* can be generated as:

(7) ZA=exp(−1RTEA)

For all atoms in the selected molecular system, the atomic ensemble energies are calculated separately to ensure that the molecular local partition function can be numerically calculated for each atomic movement in its given sampling range.

(8) ZM=ZA×ZB×ZC×⋯

By feeding the MT protocol with multiple molecular configurations, local molecular partition functions ZM can be calculated using Equation (8) for estimation of the free energy. Regarding the protein-ligand binding affinity study, conformations for both free and bound states are generated using the Monte Carlo sampling protocols followed by local minimizations. The free state molecular system includes unbound ligand and protein in the solution phase. ZL and ZP are their corresponding partition functions which are necessary for the binding free energy calculation. On the other hand, the bound state molecular system includes the protein ligand molecules in the complex form in the solution. ZPL is the bound state partition function containing all the protein-ligand binding mode energy states. In the present study we only performed the ligand conformational sampling and the protein-ligand binding mode sampling by considering the flexibility of the ligand structures and the protein binding site residues while keeping the rest of the protein geometry fixed. The protein conformational sampling is skipped because (1) the massive degrees of freedom associated with inclusion of protein flexibility will significantly increase the computational burden, while (2) having limited contributions to the computational accuracies regarding relative binding affinities studies using identical or similar protein target, due to that the ZPL values are very similar among all the test cases.

In-house programs developed in our group are introduced to perform such tasks. For the free-state calculation, the MT-CS conformational search program [55] was introduced to generate significant free-state molecular conformations with reference to the molecular flexibility. The MT-CS conformational search program generated ligand conformers using a torsion library with pre-calculated torsion energies using the GARF energy model [56], the solvation free energy was calculated using the KMTISM model [57]. The MT protocol was then applied to each ligand conformer to estimate the local partition function ZL. The ligand’s total partition function was then generated using all the MT-CS sampled configurational ensemble energies in Equation (9).

(9) ZL=∑αNL conformationZLα=ZL1+ZL2+⋯+ZLN

The Heatmap docking program [44] was employed for the bound state configuration sampling in this work. The bound-state protein-ligand complex ensemble energy is calculated using the same protocol by summing all the local partition functions.

(10) ZPL=∑αNPL conformationZPLα=ZPL1+ZPL2+⋯+ZPLN

**Figure 11:**
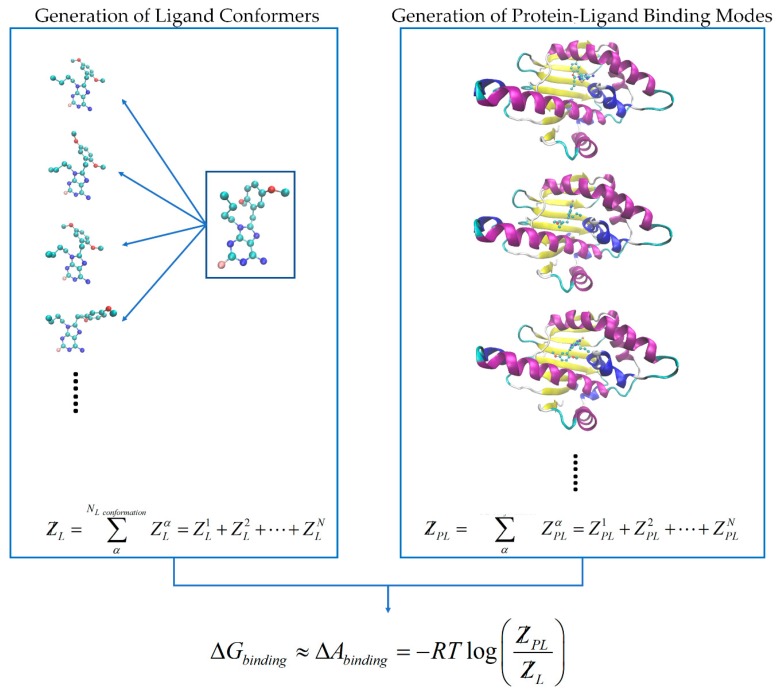
A flow chart for the ensemble energy calculation protocol employed in this work. Both bound-state and free-state ensembles were generated using the programs indicated (Heatmap docking and MT-CS). The final free energy change was then calculated using the ratio of partition functions ZPL/ZL.

The binding free energy change was then estimated by using the ratio of partition functions in bound and free states. The whole calculation protocol is also illustrated in Figure 11.

(11) ΔGbinding≈−RTlog(ZPLZL)

In this work, we utilized the MT free energy protocol briefed above for the caspase-inhibitor binding affinity study. All related codes and data can be obtained by contacting the authors for validation and review purpose only.

## 4. Conclusions

We applied our newly developed Movable Type free energy protocol to the caspase-inhibitor complexing study. Using a Monte Carlo sampling approach, the MT method generated the significant binding modes and calculated the binding free energies using the ratio of the partition functions referencing the bound state and free state protein-ligand systems. Both the large-scaling and carefully set-up small test sets were introduced to provide a comprehensive study regarding the robustness and sensitivity of the MT protocol against such complexing systems. Results revealed good agreements of the calculation predictions with the experimental binding affinities and the global minimum binding modes. Through detailed case studies, we further illustrated the MT protocol mechanism for the free energy extrapolation using a Monte Carlo based sampling method. Moreover, we also took a close look at the global minimum binding mode structures to study how minor changes in the interaction interfaces affecting the binding affinities and how with different interaction interfaces achieved similar binding affinities. Generally, this work provided us useful computational information for the binding affinity prediction using the MT protocol. Future studies including computation-experiment combinatorial research can be expected for the structural based caspase inhibitor design. We also plan to apply the MT protocol to the caspase inhibitor-related inverse docking study.

## Figures and Tables

**Table 1:** Comparison of the binding free energy calculated by Moveable Type to that obtained from experiment for the caspase-3—small molecule inhibitor test set.

PDB ID	Ligand	Ligand Mass (Da)	Experimental ΔG (kcal/mol)	Calculated ΔG (kcal/mol)
3h0e	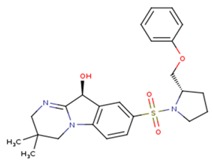	455.57	−11.11	−9.15
1gfw	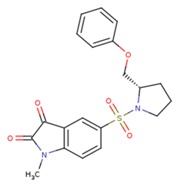	400.45	−10.66	−10.34
3dei	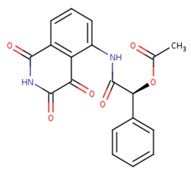	366.32	−10.08	−8.56
3dej	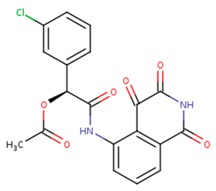	400.77	−10.90	−8.46
3dek	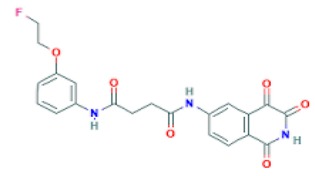	427.38	−10.32	−10.37
1nms	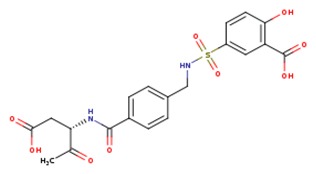	464.45	−9.13	−9.46
1re1	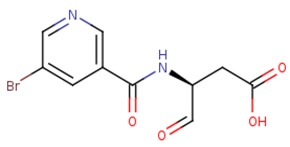	301.09	−7.099	−7.133
1rhm	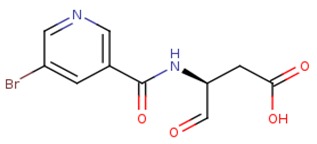	301.09	−7.849	−7.728

**Table 2:** Comparison of the binding free energy calculated by Moveable Type to that obtained from experiment for the caspase-3—peptidomimetic molecule inhibitor test set.

PDB ID	Ligand	Ligand Mass (Da)	Experimental ΔG (kcal/mol)	Calculated ΔG (kcal/mol)
1rhu	5,6,7 tricyclic peptidomimetic	638.69	−11.61	−10.88
1rhr	Cinnamic acid methyl ester	651.14	−11.04	−10.79
1rhj	Pryazinone	574.69	−10.96	−11.02
4jje	ACE-1MH-ASP-B3L-HLX-1U8 (Unnatural amino acid peptides)	838.94	−10.41	−10.90
2h5i	Ac-DEVD-Cho	504.49	−12.11	−11.18
2h5j	Ac-DMQD-Cho	535.57	−10.779	−11.06
4jr0	Ac-DEVD-CMK	552.96	−11.17	−11.26
3gjt	Ac-IEPD (Diverse P4 Residues in Peptides)	498.13	−9.23	−9.44

**Table 3:** Binding free energy calculation results by using the MT protocol against the caspase-polypeptide complexing test set.

PDB ID	Caspase Target	Peptide Ligand	Experimental ΔG (kcal/mol)	Calculated ΔG (kcal/mol)
2h5j	caspase-3	Ac-DMQD-Cho	−10.78	−11.06
2ql5	caspase-7	Ac-DMQD-Cho	−11.04	−12.72
2ql9	caspase-7	Ac-DQMD-Cho	−12.30	−12.51
2qlf	caspase-7	Ac-DNLD-Cho	−12.06	−12.26
2qlb	caspase-7	Ac-EMSD-Cho	−8.03	−8.46
2ql7	caspase-7	Ac-IEPD-Cho	−8.53	−8.16
1f1j	caspase-7	Ac-DEVD-Cho	−11.99	−10.74
2h5i	caspase-3	Ac-DEVD-Cho	−12.11	−11.18
4jr0	caspase-3	Ac-DEVD-CMK	−11.17	−11.26
3r7b	caspase-2	Ac-DVAD-Cho	−8.38	−8.42
3r5j	caspase-2	Ac-ADVAD-Cho	−9.48	−9.72
3r6g	caspase-2	Ac-VDVAD-Cho	−10.36	−10.68
3gjt	caspase-3	Ac-IEPD	−9.23	−9.44
1f9e	caspase-8	Phq-DEVD	−11.86	−10.49
4jje	caspase-3	Ac-1MH-ASP-B3L-HLX-1U8	−10.41	−10.90

## Supplementary Materials

Supplementary materials can be found at https://www.mdpi.com/1422-0067/20/19/4850/s1.

## Abbreviations

AD	Alzheimer’s Disease
MT	Movable Type
NFTs	Neurofibrillary tangles
